# Effects of a 28-day feeding trial of grain-containing versus pulse-based diets on cardiac function, taurine levels and digestibility in domestic dogs

**DOI:** 10.1371/journal.pone.0285381

**Published:** 2023-05-25

**Authors:** Chloe Quilliam, Luciana G. Reis, Yikai Ren, Yongfeng Ai, Lynn P. Weber

**Affiliations:** 1 Department of Veterinary Biomedical Sciences, University of Saskatchewan, Saskatoon, SK, Canada; 2 Department of Food and Bioproduct Sciences, University of Saskatchewan, Saskatoon, SK, Canada; University of Illinois, UNITED STATES

## Abstract

In 2018, the US Food and Drug Administration reported a link between canine dilated cardiomyopathy (DCM) and grain-free diets. Evidence to support a link has emerged, but the specific ingredients responsible and the role of taurine or other causative factors remain unclear. We hypothesized dogs fed pulse-based, grain-free diets for 28 days will show decreased macronutrient digestibility, increased fecal bile acid excretion, and reduced plasma cystine, cysteine, methionine and taurine, causing sub-clinical cardiac or blood changes indicative of early DCM. Three diets were formulated using white rice flour (grain), whole lentil (grain-free), or wrinkled pea (grain-free) and compared to the pre-trial phase on a commercial grain-based diet. After 28 days of feeding each diet, the wrinkled pea diet impaired stroke volume and cardiac output, increased end-systolic ventricular diameter and increased plasma N-Terminal Pro-B-type Natriuretic Peptide (NT-ProBNP), albeit in a sub-clinical manner. Digestibility of some macronutrients and sulphur-containing amino acids, excluding taurine, also decreased with pulse-based compared to grain-based diets, likely due to higher fiber levels. Plasma taurine levels were unchanged; however, plasma methionine was significantly lower after feeding all test diets compared to the commercial diet. Overall, DCM-like changes observed with the wrinkled pea diet, but not lentil diet, after only 4 weeks in a breed not known to be susceptible support a link between pea-based diets and canine nutritionally-mediated DCM.

## Introduction

Grain-free diets in the pet food industry are diets that do not use traditional grains such as wheat, corn and rice flours. Instead, these diets use pulses such as peas, chickpeas and lentils, potatoes and other ingredients as their carbohydrate source [1]. Pulses are feed ingredients that have been used in many dog foods over the past two decades [2]. Pulses have been commonly incorporated into human and pet diets as economical ingredients that contain high levels of protein, fiber, oligosaccharides, vitamins and minerals [3]. Pulse crops are also slowly digested due to their relatively high dietary fiber and amylose levels, ideal for low glycemic responses and increased satiety in dogs [4–6].

In July 2018, the US Food and Drug Administration (FDA) reported that there was a link between canine dilated cardiomyopathy (DCM) and diets containing peas, lentils, other legumes, sweet potatoes or potatoes in dog breeds not known to be genetically susceptible to this disease [6]. A subsequent study identified peas and, to a lesser extent lentils, as distinguishing ingredients between diets associated with nutritional DCM among the top 16 diets reported by the FDA compared to diets not associated with DCM [7]. Though peas and other pulses are high in plant proteins, they lack taurine, which is only obtained through animal protein sources. However, several recent studies do not support an early hypothesis that low dietary taurine is the causal link to diet-induced adverse changes in cardiac function or DCM in dogs since whole blood or plasma taurine levels were reported above deficiency levels in most dogs [8–10]. Some studies have reported evidence to support a link between low blood or plasma levels of taurine and DCM in dogs [11, 12], while another failed to find a link [13]. Moreover, changing to either grain-inclusive diets without pulses and potatoes or with taurine supplementation have all been reported to improve cardiac function and DCM indicators in dogs, some of which had pre-existing disease and somebreeds with genetic susceptibility [9, 10, 14]. However, another study reported no notable adverse change in blood taurine when healthy Labrador Retrievers were fed a grain-free, pea- and lentil-containing diet for 30 days [15], while another that fed a vegan, high pea-content diet to healthy dogs of multiple breeds for 12 weeks did report adverse changes consistent with early DCM [16]. Thus there remains a need for further investigations in this area. Additionally, pulses contain a limited amount of the amino acids cysteine and methionine [3]. Cysteine and methionine are both used to synthesize taurine via the transsulfuration pathway in the dog [12]. The limited sulphur-amino acid content in pulse-based diets could further contribute to the taurine-deficiency which in turn may link to nutritional DCM in dogs.

DCM in dogs is a myocardial disorder that is accompanied by left ventricular systolic dysfunction (ejection fraction < 40% accompanied by increased end-diastolic and systolic volumes), reduced left ventricular wall thickness and ventricular chamber dilation [17]. Primary DCM is a genetically-based disease where certain pre-disposed breeds including Doberman Pinschers and Boxers do not show a link to taurine deficiency. In contrast, Golden Retrievers appear to be predisposed to DCM that is secondary to taurine deficiency [12]. Taurine holds high importance for myocardial function, but also plays a role in the conjugation of bile acids such as taurocholic acid [18]. Taurine depletion has been associated with increased dietary fiber [19], but is also associated with low dietary protein through enterohepatic losses [18]. Improvement has been reported when dogs with secondary or nutritional DCM are switched to diets lacking pulse ingredients, especially when DCM was subclinical [9, 10, 13, 14]. During sub-clinical stages of nutritional DCM, dogs remain asymptomatic, but begin having echocardiographic changes demonstrating structural abnormalities such as increased ventricular chamber volume and size [17]. Elevated levels of the blood markers N-Terminal Pro-B-type Natriuretic Peptide (NT-ProBNP) and cardiac troponin I are additional screening measures that can be performed as they increase with cardiac changes associated with both types of DCM [17, 20]. Breeds such as Dobermans and Boxers are commonly diagnosed with genetic DCM [8, 10, 13]. Despite this, there has been an increased observance of DCM in dogs of all sizes, but particularly small breeds that alerted veterinarians to a possible diet link [8, 10, 14]. Establishing DCM links to taurine deficiency and a high pulse diet require further investigation. A study previously conducted in our lab looked at the effects of feeding pulse-based, grain-free diets for 7 days in laboratory Beagles did not detect any changes in plasma amino acids, taurine or cardiac function [21]. However, this may be expected due to the short duration of the study and a longer feeding study is warranted.

The following study aims to further evaluate the effects of pulse-inclusive diets in dogs with a specific focus on macronutrient digestibility, bile acid excretion, plasma amino acid levels (cystine, cysteine, methionine and taurine) and blood markers of overall health as possible links to adverse changes in cardiac function or cardiac biomarkers. Specifically, this study investigated whether dietary fiber, amylose or oligosaccharide content of pulse-inclusive diets were potential links to nutritional DCM. For this study, it was hypothesized that in dogs, feeding pulse-based, grain-free diets for a longer period of 28 days will cause a decrease in macronutrient digestibility, an increase in the excretion of fecal bile acids, and a decrease in plasma levels of cystine, cysteine, methionine and taurine, resulting in sub-clinical cardiac and blood changes consistent with early signs of DCM. From our previous 7-day study that examined 6 diets [21], the pulse diets with the highest and lowest fiber/amylose content (wrinkled pea and lentil, respectively) were chosen for the current 28-day study and compared to a rice-based diet, all formulated at 15% enzymatic starch. Results from these lab-made diets were compared to values from dogs fed a commercial, grain-containing diet during the pre-trial phase.

## Materials and methods

All procedures and handling of the dogs were conducted following protocols approved by the University of Saskatchewan’s Animal Research Ethics Board according to guidelines that were established by the Canadian Council on Animal Care (Animal Utilization Protocol #20190055).

### Animals

Adult Beagle dogs (n = 8; 4 spayed females, 4 neutered males, 9.4 ± 0.4 kg, 3–5 years old) were obtained from certified scientific breeders (Marshall Bioresources, North Rose, NY, USA and King Fisher International, Stouffville, ON, Canada). The research Beagles were housed at the Animal Care Unit in the Western College of Veterinary Medicine at the University of Saskatchewan (Saskatoon, SK, Canada). The Beagles were group-housed during the day in a large enclosure allowing for daily socialization but were individually kenneled during feedings and overnight. The dogs were walked and socialized on a daily basis. The Beagles were also provided regular health examinations, deworming and routine vaccinations from veterinarians to ensure optimal health.

### Diets

During the pre-trial phase, dogs were fed a commercial, grain-based diet and values prior to commencing the test diets are included for comparison to the lab-made diets. The lab-made diets formulated for this study included one control (rice; a grain-containing diet) and two pulse-based diets (red lentil (CDC Maxim) and wrinkled pea (Amigold variety); both grain-free diets). All diets for this study were formulated to contain 15% enzymatic starch (total enzymatic starch determined with an R-BIOPHARM Enzymatic BioAnalysis) using flours that were locally obtained (flour proximate analyses shown in the Table A of S1 Table). A non-digestible Celite marker was also incorporated into all diets at 1% to determine the total tract apparent digestibility of each diet. This marker was measured as acid-insoluble ash in the proximate analyses of both diet and feces [22]. Research diets were formulated using the software Creative Concept 5 (Creative Formulation Concepts, Pierz, MN, USA). All diets were formulated to meet the nutritional requirements for canine adult maintenance (See Table B of S1 Table for diet formulations) based on recommendations made by The Association of American Feed Control Officials (AAFCO). Feed ingredients for all diets were from both local and commercial sources as required and extruded into a dry kibble format using a laboratory-scale, co-rotating, twin-screw extruder (TwinLab-F 20/40, C. W. Brabender Instruments Inc., South Hackensack, NJ, USA) at the University of Saskatchewan (Food Science Laboratory, Saskatoon, SK, Canada). Extrusion conditions for all diets remained constant and are described in the Table C of S1 Table. After extrusion diets were air dried for 48 hours and then vacuum coated with oil and chicken fat at the Canadian Feed Research Centre (North Battleford, SK, Canada). Once all diets were complete individual samples were sent to multiple analytical laboratories for analyses. Proximate and amino acid analyses (Central Testing Laboratory Ltd., Winnipeg, MB, Canada) were performed according to the AOAC standards [23]. Since cysteine readily oxidizes to form a dimer called cystine, all diet and fecal levels of cysteine are reported as cystine. Dry matter of all samples was determined by oven-drying the sample, while crude protein was determined using the Kjeldahl method. In addition, non-fiber carbohydrate and fat were determined through acid-hydrolysis solvent extraction. All diet samples were also subjected to fiber analyses to determine the soluble, insoluble and total high molecular weight dietary fiber (HMWDF) content (Eurofins, Toronto, ON, Canada) according to the AOAC 991.43 methods [23]. Eurofins was also contracted to determine the oligosaccharide content in each diet using internal high performance liquid chromatogrqaphy-based laboratory methods. Specifically raffinose, stachyose and verbascose were measured since they are the most abundant oligosaccharides.

### Feeding of diets

Research Beagles were fed twice daily, weighed weekly and assessed for body condition using a 9-point scale [24]. During the pre-trial phase, the dogs were fed a standard commercial, grain-containing diet for a minimum of 28 days (Purina Pro Plan® Adult Complete Essentials Shredded Blend Beef & Rice Dry Dog Food; See Table D of S1 Table for diet details) which was used to establish baseline values before starting the research study. During this period, each dog was fed individual feed portions that were calculated to maintain ideal body weight and body condition (score of 4–5 on a 9-point scale). Once the feeding trial started, isocaloric measurements of each test diet were calculated and measured for each dog, as determined in the pre-trial phase, and used throughout the entire feeding trial without any further adjustment of portion size.

The dogs were fed each test diet for 28 days continuously. Diet types were blinded to the researchers and all dogs were fed the same diet within the same time periods. The rice diet was fed twice in two separate 28-day feeding periods. Thus, the rice diet was used as a control as well as a washout between feeding the lentil and wrinkled pea diets to ensure that results were reliable and repeatable. Results from the two rice feeding periods did not statistically differ from each other. Thus the values from the two feeding periods were averaged and the mean value used in all graphs and statistical analyses. The feeding order that was performed was commercial-rice-lentil-rice-wrinkled pea. Similar to the pre-trial phase, all dogs were fed individually measured portions of test diet twice daily, weighed weekly and assessed weekly for body condition using a 9-point scale.

### Digestibility testing

Total tract apparent digestibility was determined using feces that were collected on days 27 and 28 of each feeding period. Once fecal samples were collected, they were frozen at -20˚C until they were dried at 65˚C for 72 hours. Once all samples were dried, they were sent to an analytical lab to assess nutrient excretion (Central Testing Laboratory Ltd., Winnipeg, MB, Canada). Total tract apparent digestibility was calculated using the formula:

$$Apparent\;Digestibility\;(\%)=\{1-\frac{\left(\%\;Nutrient\;in\;Feces\right)}{\left(\%\;Nutrient\;in\;Diet\right)}\times \frac{\left(\%\;Indicator\;in\;Diet\right)}{\left(\%\;Indicator\;in\;Feces\right)}\}\times 100$$

In addition, total bile acid analyses were conducted on the feces using a commercial kit as per manufacturer instruction (Total Bile Acid Assay Kit, Cell Biolabs Inc. San Diego, CA, USA). This assay kit used a colorimetric enzyme-driven reaction while bile acids are incubated with the presence of 3-alpha hydrocysteroid dehydrogenase and thio-NADH.

### Whole blood and plasma analyses

Leading into day 28 of feeding each test diet, dogs were fasted overnight and 8.0 mL of blood was collected the following morning from the jugular vein. Three mL of fasted blood was collected and sent to an external laboratory for the performance of a complete blood count (CBC) and Chemistry Panel (Prairie Diagnostic Services, Saskatoon, SK, Canada), while 5 mL of blood was collected into an EDTA tube and centrifuged at 2200 RPM to collect plasma. Once plasma was obtained, it was stored at -80˚C until assayed. Once all plasma samples were collected from the dogs for all diets, they were sent to a contract analytical laboratory to assay for cysteine, cystine, methionine and taurine (The Metabolomics Innovation Centre, Edmonton, AB, Canada). Quantitation of these amino acids was determined using Ultra-high Pressure Liquid Chromatography-Multiple Reaction Monitoring- Mass Spectrometry methodology. Plasma was also used to determine NT-ProBNP (catalogue# EKX-DZRREA) and cardiac troponin I (hs-cTnI, High Sensitivity Cardiac Troponin I, catalogue # EKX-B817F1) using canine-specific commercially available ELISA kits as per instructions (Nordic Biosite, Täby, Sweden). Both kits reported inter- and intra-assay variabilities of 8–10%. Any sample results below the detection limit stated in the assay kit was given a value of zero.

### Cardiac assessment

Prior to performing this experiment, the researcher (CQ) collecting the data was instructed in echocardiography techniques by an experienced echocardiographer and logged a minimum of 200 hours of practice. Prior to experiments, repeated measurements were taken of the same dogs to ensure technique was robust enough to produce repeatable cardiac results (See Table E of S1 Table). On day 28 of feeding each test diet, a cardiac assessment was performed on each dog. Each dog was conscious and not sedated during all cardiac procedures. The cardiac assessment was performed and analyzed by one researcher. To begin the cardiac assessment, multiple blood pressure measurements were taken using a high-definition canine/feline oscillometer (VET HDO High Definition Oscillometer, Babenhausen, Germany) at the base of the tail. An average of three readings per dog were taken and used to determine the systolic and diastolic pressures. After blood pressure measurements were taken, dogs were shaved creating windows for echocardiography. Echocardiography was performed with a Sonosite Edge II ultrasound (Fujifilm Sonosite Canada, Markham, ON, Canada) using a P10x transducer (8–4 Hz). An average of two values from the electrocardiogram taken simultaneously during cardiac measurements in two different cine loops that were used to determine heart rate. All linear cardiac measurements and volume values were taken from a minimum of two independent cardiac cine loops for each measurement and averaged values used for each dog. Averaged values were normalized to each dog’s body weight for volumes measured using Simpson’s method of discs or to an exponent of body weight for linear measurements in M-mode according to methodology found within literature [25, 26]. The left ventricle (LV) was imaged from parasternal long axis 2- and 4-chamber apical views and used to calculate LV end-diastolic volume (EDV), end-systolic volume (ESV), stroke volume (SV) and cardiac output (CO). Ejection fraction was calculated as the ratio of SV to EDV from the biplane Simpson’s rule values. For linear LV measurements, a right parasternal short axis view was used for M-mode imaging of the LV at the level of the papillary muscles to measure LV inner diameter (LVID) at systole and diastole.

### Data handling and statistics

All raw data can be found in S1 Text. All data were tested for normality and outliers using the Kolomogorov-Smirnov test, Q-Q plots and box plots. Parametric data was expressed as mean ± standard error of the mean (SEM) while non-parametric data was expressed as median and range. All data from both rice feeding periods were subjected to paired t-tests to determine if they were statistically different (p < 0.05). It was determined that the data was not significantly different (p > 0.05) between the two rice feeding periods and all rice data for each dog was averaged and used for further statistical analyses. Depending on the normality of the data either a repeated measures, one-way ANOVA or a repeated measures one-way ANOVA on ranked data was then performed followed by post-hoc Tukey’s tests where significance was achieved. Differences were considered to be statistically significant at p ≤ 0.05. Analyses were performed using Systat 12.0 (Systat Software Inc. San Jose, CA, USA).

## Results

### Proximate, fiber and amylose analyses of diets

All 3 formulated diets and the commercial diet contained similar levels of crude protein and crude fiber (Table 1). Enzymatic starch was designed to be constant in the lab-made test diets, while the commercial diet had a larger content of this type of starch. Despite this constant enzymatic starch, amylose content varied and was highest in the wrinkled pea diet (Table 1). In contrast, crude fat of all diets showed the most variation among diets of all the macronutrients (Table 1). While crude fiber was relatively constant among diets, the rice diet had the lowest levels of both non-fiber carbohydrate and total digestible nutrients among the diets (Table 1). Total and insoluble high molecular weight dietary fiber (HMWDF) fractions were higher in the two pulse-inclusive diets (lentil and wrinkled pea) compared to the rice diet, while soluble fiber was highest in the rice diet (Table 1). The commercial and rice test diet had no detectable levels of oligosaccharides (raffinose, stachyose and verbascose; Table 1). In contrast, the lentil diet contained detectable levels of stachyose and verbascose, but not raffinose, while the wrinkled pea diet contained the highest levels of all three oligosaccharides (Table 1).

**Table 1:** Proximate analyses of test diets. Proximates, fiber, oligosaccharide and amylose content of the commercial grain-containing diet and different formulated test diets after extrusion and fat coating are shown. Diets ordered from left to right in order of increasing total high molecular weight dietary fiber (HMWDF) content.

	Commercial Diet	Rice Diet	Lentil Diet	Wrinkled Pea Diet
DM	93.7	90.9	90.6	87.8
Crude Protein ^a^	30.9 (73.0)	33.5 (88.0)	31.8 (81.0)	31.0 (78.8)
Crude Fiber ^b^	0.86	7.47	7.39	7.40
Fat ^c^	15.1 (35.7)	20.3 (53.3)	18.5 (47.1)	18.1 (46.1)
Ash ^d^	7.11	0.96	7.51	7.28
Non-Fiber Carbohydrate ^e^	45.1 (106.4)	26.8 (70.5)	33.9 (86.3)	35.4 (90.0)
Total Digestible Nutrients ^f^	83.1	74.3	76.2	76.4
Metabolizable Energy ^g^	4235	3801	3928	3930
Soluble HMWDF ^h^	0.5	0.8	0.6	0.7
Insoluble HMWDF ^h^	5.0	14.8	16.1	16.5
Total HMWDF ^h^	5.5	15.6	16.7	17.2
Raffinose ^i^	<0.2	<0.2	<0.2	0.6
Stachyose ^i^	<0.2	<0.2	0.9	1.4
Verbascose ^i^	<0.2	<0.2	0.4	2.3
Enzymatic Starch ^j^	24.5 (57.9)	15.8 (41.4)	15.3 (38.9)	15.9 (40.5)
Amylose content ^k^	8.1	4.6	6.7	12.8

DM = dry matter; All % values are relative to dry matter excluding Metabolizable Energy (kcal/kg). Values in brackets are select proximate values expressed relative to Metabolizable Energy (g/1000 kcal).

^a^ Determined by Central Testing Laboratory Ltd. (Winnipeg, MB, Canada), following AOAC Method 990.03(M)

^b^ Determined by Central Testing Laboratory Ltd. (Winnipeg, MB, Canada), following Crude Fiber Method by Ankom Technology (2017), based on AOCS Ba 6a-05.

^c^ Determined by Central Testing Laboratory Ltd. (Winnipeg, MB, Canada), following AOCS Method AM 5–04.

^d^ Determined by Central Testing Laboratory Ltd. (Winnipeg, MB, Canada), following AOAC Method 942.05.

^e^ Determined using the equation for Non-Fiber Carbohydrates (NFC, Dry Matter): NFC (%) = 100-[(%Dry Matter/100)+%Protein+%Fat+%Ash+%Crude Fiber]

^f^ Determined by Central Testing Laboratory Ltd. (Winnipeg, MB, Canada) using the equation for Total Digestible Nutrients (TDN, Dry Matter): TDN (%) = 100-[(%Dry matter/100)+%Ash+%Crude Fiber]-8

^g^ Determined by Central Testing Laboratory Ltd. (Winnipeg, MB, Canada) using the ME equation for swine: (kcal/kg) = 4151-(122*Ash)+(23*Crude Protein)+(38*Fat)-(64*Crude fiber)*(1.003-(0.0021* Crude Protein)) by Central Testing Laboratory Ltd. (Winnipeg, MB, Canada)

^h^ Determined by Eurofins (Toronto, ON, Canada) using AOAC 991.43 dietary fiber method.

^i^ Determined by Eurofins (Toronto, ON, Canada) using an in-house high performance liquid chromatography method

^j^Determined by Central Testing Laboratory Ltd. (Winnipeg, MB, Canada), R-Biopharm Test kit (UV Method)

^k^ Determined using an iodine colorimetric method of [27].

The wrinkled pea diet had lower methionine levels than the rice diet, while both the lentil and wrinkled pea diets had lower cystine+methionine levels (Table 2). Taurine levels showed little variation among diets (Table 2).

**Table 2:** Sulphur amino acids in diets. Measured content of cystine, methionine, cystine+methionine and taurine in the 3 different test diets formulated at 15% enzymatic starch with variable amounts of fiber, fed for 28 days. For comparison, the recommended dietary minimum amino acid levels by the American Association of Feed Controllers (AAFCO) are also shown.

	Commercial Diet	Rice Diet	Lentil Diet	Wrinkled Pea Diet	AAFCO minimum
Cystine ^a^	0.32 (0.76)	0.23 (0.61)	0.20 (0.51)	0.26 (0.66)	-^b^
Methionine ^a^	0.43 (1.02)	0.59 (1.55)	0.36 (0.92)	0.32 (0.81)	0.33 (0.83)
Cystine+Methionine ^a^	0.75 (1.77)	0.82 (2.16)	0.56 (1.43)	0.58 (1.48)	0.65 (1.63)
Taurine ^a^	0.08 (0.19)	0.10 (0.26)	0.08 (0.20)	0.09 (0.23)	- ^b^

Diets ordered from left to right in order of increasing total high molecular weight dietary fiber (HMWDF) content.

All values are expressed as % dry matter or values in brackets indicating g/Mcal.

^a^ Determined by Central Testing (Winnipeg, MB, Canada) using ultra-high pressure liquid chromatography and ninhydrin detection.

^b^ There is no requirement for these individual amino acids in adult dogs.

### Total tract apparent digestibility

Total tract apparent digestibility of crude protein varied across the diets, being significantly higher in the rice diet compared to both the lentil and wrinkled pea diets (Table 3). Similarly, fat digestibility was highest and significantly different in the rice diet compared to the wrinkled pea diet (Table 3). The wrinkled pea diet had significantly lower starch digestibility compared to both the rice and lentil diets (Table 3). Non-fiber carbohydrate digestibility and total digestible nutrients were significantly lower in the lentil diet compared to the wrinkled pea and rice diets, respectively (Table 3). The lentil diet had the lowest cystine digestibility compared to the other two diets (Table 3), while both the lentil and the wrinkled pea diets had lower methionine digestibility when compared to the rice diet (Table 3). Similarly, both pulse-inclusive diets also had significantly lower cystine+methionine digestibility when compared to the rice diet (Table 3). No significant differences were observed in the total tract apparent digestibility of taurine among the rice, lentil or pea diets after 28 days of feeding.

**Table 3:** Digestibility of test diets. Total tract apparent digestibility analyses of different test diets formulated at 15% enzymatic starch with variable amounts of fiber, fed for 28 days. Diets ordered from left to right in order of increasing total high molecular weight dietary fiber (HMWDF) content.

	Rice Diet	Lentil Diet	Wrinkled Pea Diet	p-Value
Crude Protein **	86.4 (83.6–87.7)^a^	78.0 (74.3–80.1)^b^	78.9 (60.0–93.4)^b^	0.008
Fat **	98.9 (96.0–99.4)^a^	97.0 (90.7–98.5)^a,b^	96.4 (79.2–99.0)^b^	0.018
Starch *	99.3 ± 0.5^a^	99.7 ± 0.2^a^	92.7 ± 1.6^b^	<0.001
Non-Fiber Carbohydrate *	62.2 ± 1.8^a,b^	54.6 ± 1.6^b^	66.8 ± 3.6^a^	0.018
Total Digestible Nutrients **	82.5 (79.0–86.7)^a^	73.5 (71.1–78.0)^b^	77.0 (74.2–93.0)^a,b^	<0.001
Cystine *	81.8 ± 0.9^a^	69.1 ± 2.7^b^	78.7 ± 2.4^a^	0.003
Methionine *	91.7 ± 0.7^a^	82.7 ± 1.6^b^	79.6 ± 2.0^b^	<0.001
Cystine+Methionine *	88.9 ± 0.7^a^	77.9 ± 1.7^b^	79.2 ± 2.1^b^	<0.001
Taurine **	100.0 (100.0–100.0)	100.0 (95.1–100.0)	100.0 (95.1–100.0)	0.790

All values are expressed as % of total tract apparent digestibility.

Data is shown as Mean ± SEM or Median (range); n = 8 dogs. Different letters within a row indicate significant differences among diets using Tukey’s post-hoc analysis (p < 0.05) after significant p-values reported in one-way repeated measures ANOVA* and Friedman’s one-way ANOVA on ranked data**.

Protein, fat, non-fiber carbohydrate and total digestible nutrient determined by Central Testing (Winnipeg, MB Canada) as described in Table 1. Diet and fecal amino acids and starch were determined by Central Testing (Winnipeg, MB, Canada) using ultra-high pressure liquid chromatography with ninhydrin detection and enzymatically using UV detection, respectively.

Commercial diet was not included for total tract apparent digestibility analyses due to not having a digestible marker in it.

### Bile acid analysis

**Fig 1:**
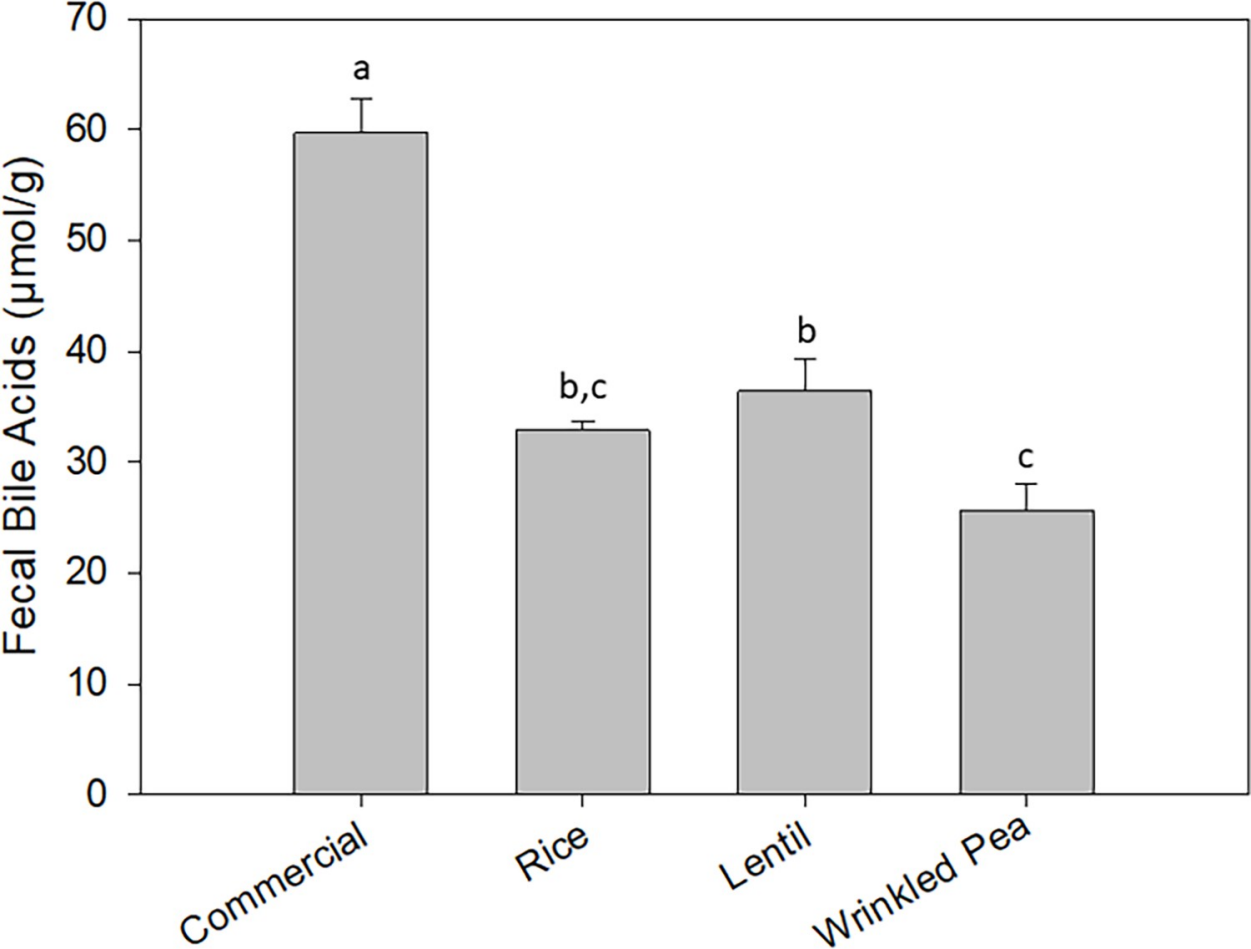
Fecal bile acid content (μmol/g) from dogs after 28 days of feeding each test diet. Diets ordered from left to right in order of increasing total high molecular weight dietary fiber (HMWDF) content. Data is shown as Mean ± SEM; n = 8 dogs. Different letters indicate significant differences using Tukey’s post-hoc analysis (p < 0.05) after one-way repeated measures ANOVA.

Fecal bile acid content significantly differed among the different diets, with the commercial grain-containing diet having the highest excretion and the wrinkled pea diet having the lowest (Fig 1).

#### Complete Blood Count (CBC) and blood chemistry

The CBC in dogs had no significant differences among diets for both white blood cells and platelets (Table 4). In contrast, dogs on the commercial diet had significantly higher RBC count than the lab-made rice and pea diets (Table 4). Despite the differences, all values for the dogs on all diets in the CBC fell within the reference range.

**Table 4:** Complete blood count (CBC). CBC in dogs fed different grain-containing and grain-free diets for 28 days. Diets ordered from left to right in order of increasing total high molecular weight dietary fiber (HMWDF) content.

	Commercial Diet	Rice	Lentil	Wrinkled Pea	p-Value	Reference Range
White blood cells (x 10^9^/L)*	6.44 ± 0.45	5.67 ± 0.37	6.20 ± 0.39	5.89 ± 0.38	0.064	4.9–15.4
Platelets (x 10^9^/L)**	250 (192–487)	233 (189–463)	235 (202–477)	259 (162–477)	0.270	117–418
RBC (x 10^12^/L)*	6.97 ± 0.24^a^	6.49 ± 0.12^b^	6.64 ± 0.12^a,b^	6.51 ± 0.12^b^	0.022	5.80–8.50

Data is shown as Mean ± SEM or Median (range); n = 8 dogs. Different letters within a row indicate significant differences among diets using Tukey’s post-hoc analysis (p < 0.05) after significant p-values reported in one-way repeated measures ANOVA* and Friedman’s one-way ANOVA on ranked data**.

Red blood cells (RBC).

Blood parameters of hepatic function from the blood chemistry performed after feeding each diet for 28 days are shown in Table 5. Serum cholesterol was significant higher in dogs on the commercial diet when compared to the rice and lentil diets, but all individual values still fell within the reference range for adult dogs (Table 5). Average total bilirubin for dogs on the lentil diet fell below the reference range, but no significant differences among diets were detected (Table 5). Direct bilirubin had significant differences in dogs among the diets, with the dogs consuming the commercial diet having the lowest values compared to those from the rice diet (Table 5). Indirect bilirubin levels in dogs after eating all test diets fell within reference range. However, indirect bilirubin values after dogs ate all three test diets were significantly lower than after they ate the commercial diet (Table 5). Similarly, alkaline phosphate (ALP) in the dogs was highest when the commercial diet was eaten compared to when all three lab made diets were consumed. All individual gamma-glutamyl transferase (GGT) and creatine kinase (CK) levels fell within reference range for the dogs on all diets. For GGT, the values after the rice diet was fed were significantly higher than after the lentil diet, while for CK, the lentil diet was significantly higher than after all the other diets. Total protein and globulin levels for most dogs on all diets also fell slightly below reference range. Total protein was significantly higher after dogs ate the lentil compared to the rice diet while globulin was significantly higher after dogs ate the commercial diet compared to either the rice or wrinkled pea diets (Table 5). Albumin was unaltered by diet, while the albumin:globulin (A:G) ratio was significantly lower after dogs ate the commercial diet compared to the rice and lentil diets (Table 5).

**Table 5:** Hepatic function. Blood parameters of hepatic function in dogs fed different grain-containing and grain-free diets for 28 days. Diets ordered from left to right in order of increasing total high molecular weight dietary fiber (HMWDF) content.

	Commercial Diet	Rice Diet	Lentil Diet	Wrinkled Pea Diet	p-Value	Reference Range
Cholesterol (mmol/L)**	5.59 (3.93–6.64)^a^	4.53 (3.23–5.54)^b^	4.29 (2.93–5.26)^b^	4.82 (2.84–5.58)^a,b^	<0.001	2.70–5.94
Total Bilirubin (μmol/L)*	1.34 ± 0.15	1.17 ± 0.09	0.96 ± 0.11	1.03 ± 0.09	0.060	1.0–4.0
Direct Bilirubin (μmol/L)*	0.44 ± 0.03^a^	0.65 ± 0.05^b^	0.55 ± 0.07^a,b^	0.64 ± 0.05^a,b^	0.035	0.0–2.0
Indirect Bilirubin (μmol/L)*	0.90 ± 0.15^a^	0.52 ± 0.06^b^	0.41 ± 0.29^b^	0.39 ± 0.09^b^	0.001	0.0–2.5
ALP (U/L)*	49.3 ± 7.3^a^	33.9 ± 4.5^b^	36.1 ± 5.3^b^	32.4 ± 4.9^b^	<0.001	9.0–90
GGT (UL)*	1.8 ± 0.4^a,b^	2.0 ± 0.3^a^	0.4 ± 0.4^b^	1.1 ± 0.5^a,b^	0.026	0.0–8.0
ALT (U/L)**	22.0 (19.0–27.0)^a,b^	20.8 (17.0–23.5)^b,c^	23.0 (22.0–33.0)^a^	22.5 (19.0–40.0)^b,c^	<0.001	19.0–59.0
GLDH (U/L)*	3.6 ± 0.2	3.6 ± 0.3	3.1 ± 0.4	3.3 ± 0.5	0.520	0.0–7.0
CK (U/L)*	137 ± 10^a^	126 ± 5^a^	228 ± 17^b^	112 ± 8^a^	<0.001	51.0–418.0
Total Protein (g/L)**	52.5 (46.0–59.0)^a,b^	51.8 (45.5–54.5)^a^	54.0 (46.0–58.0)^b^	52.0 (42.0–55.0)^a, b^	0.011	55.0–71.0
Albumin (g/L)**	34.0 (27.0–38.0)	34.0 (29.0–36.5)	35.0 (29.0–37.0)	33.0 (26.0–38.0)	0.052	32.0–42.0
Globulin (g/L)*	19.8 ± 0.7^a^	18.1 ± 0.4^b^	18.9 ± 0.7^a,b^	18.5 ± 0.6^b^	<0.001	20.0–34.0
A:G	1.7 ± 0.1^a^	1.9 ± 0.1^b^	1.8 ± 0.1^b^	1.8 ± 0.1^a,b^	0.006	1.06–1.82

Data is shown as Mean ± SEM or Median (range); n = 8 dogs. Different letters within a row indicate significant differences among diets using Tukey’s post-hoc analysis (p < 0.05) after significant p-values reported in one-way repeated measures ANOVA* and Friedman’s one-way ANOVA on ranked data**.

Alkaline phosphate (ALP), gamma-glutamyl transferase (GGT), alanine aminotransferase (ALT), glutamate dehydrogenase (GLDH), creatinine kinase (CK), albumin:globulin (A:G).

All individual blood electrolyte values for the dogs fell within reference range, except for calcium levels when dogs consumed the commercial diet (Table 6) where the median value was above reference range. Blood calcium was significantly higher after feeding the commercial diet compared to all three lab made diets. Blood sodium levels were unchanged by diet, while potassium was significantly higher after dogs ate the wrinkled pea diet compared to the rice diet (Table 6). The Na:K ratio was significantly higher in dogs after eating the rice diet compared to either the lentil or wrinkled pea diets (Table 6). Bicarbonate levels were significantly lower after dogs ate the rice diet compared to the lentil diet, but anion gap was not significantly different with diet (Table 6). Blood phosphorus and magnesium levels were all within reference range, but were significantly higher in dogs after consuming the lentil diet compared to both the rice and wrinkled pea diets or compared to only the rice diet, respectively (Table 6).

**Table 6:** Electrolytes. Blood electrolytes in dogs fed different grain-containing and grain-free diets for 28 days. Diets ordered from left to right in order of increasing total high molecular weight dietary fiber (HMWDF) content.

	Commercial Diet	Rice	Lentil	Wrinkled Pea	p-Value	Reference Range
Sodium (mmol/L)*	148.0 ± 0.6	147.4 ± 0.5	147.6 ± 0.6	146.6 ± 0.8	0.270	140.0–153.0
Potassium (mmol/L)*	4.6 ± 0.1^a,b^	4.3 ± 0.1^b^	4.6 ± 0.1^a,b^	4.6 ± 0.1^a^	0.024	3.80–5.60
Na:K*	32.4 ± 0.6^a,b^	34.3 ± 0.6^a^	32.0 ± 0.5^b^	31.8 ± 0.6^b^	0.006	28.0–38.0
Chloride (mmol/L)*	115.1 ± 0.7^a,b^	113.5 ± 0.5^a,b^	113.1 ± 0.8^b^	112.8 ± 0.9^b^	0.012	105.0–120.0
Bicarbonate (mmol/L)*	20.0 ± 0.9^a^	19.8 ± 0.3^b^	22.1 ± 0.4^a^	21.1 ± 0.6^a,b^	0.026	15.0–25.0
Anion Gap (mmol/L)*	17.50 ± 0.42	18.50 ± 0.48	17.25 ± 0.31	17.50 ± 0.50	0.055	12.0–26.0
Calcium (mmol/L)**	3.4 (3.1–3.5)^a^	2.5 (2.3–2.5)^b^	2.5 (2.4–2.5)^b^	2.5 (2.3–2.5)^b^	0.002	1.91–3.03
Phosphorus(mmol/L)*	1.26 ± 0.04^a,b^	1.19 ± 0.04^a^	1.40 ± 0.04^b^	1.25 ± 0.05^a^	0.004	0.63–2.41
Magnesium (mmol/L)*	0.83 ± 0.01^b,c^	0.79 ± 0.01^a,b^	0.85 ± 0.01^c^	0.82 ± 0.02^a,b,c^	0.005	0.70–1.16

Data is shown as Mean ± SEM or Median (range); n = 8 dogs. Different letters within a row indicate significant differences among diets using Tukey’s post-hoc analysis (p < 0.05) after significant p-values reported in one-way repeated measures ANOVA* and Friedman’s one-way ANOVA on ranked data**.

Sodium:Potassium (Na:K)

Blood parameters of kidney function, digestive enzymes and blood glucose are shown in Table 7. All values fell within reference range and neither creatinine nor lipase were significantly affected by diet (Table 7). Urea was significantly higher after dogs consumed the rice diet compared to the wrinkled pea diet (Table 7). Amylase levels were significantly higher in dogs after consuming the commercial diet compared to after the rice and wrinkled pea diets, as shown in Table 7. Blood glucose levels of the dogs also significantly differed and was significantly lower after dogs were fed the lentil diet compared to all other diets tested (Table 7).

**Table 7:** Other hematological values. Blood parameters of kidney function, digestive enzymes and blood glucose in dogs fed different grain-containing and grain-free diets for 28 days. Diets ordered from left to right in order of increasing total high molecular weight dietary fiber (HMWDF) content.

	Commercial Diet	Rice	Lentil	Wrinkled Pea	p-Value	Reference Range
Urea (mmol/L)**	5.8 (5.1–6.4)^b,c^	7.4 (6.7–8.9)^a^	6.8 (5.5–9.5)^a,b^	5.6 (4.3–6.6)8^c^	<0.001	3.5–11.4
Creatinine (μmol/L)**	60.5 (48.0–75.0)	69.3 (44.5–93.0)	60.5 (48.0–103.0)	58.0 (51.0–77.0)	0.700	41.0–121.0
Amylase (U/L)*	531.4 ± 56.8^c^	457.8 ± 46.9^a,b^	482.1 ± 41.1^b,c^	468.8 ± 40.3^a,b^	0.011	343.0–1375.0
Lipase (U/L)*	55.1 ± 8.2	47.4 ± 4.8	51.0 ± 6.9	55.1 ± 5.2	0.190	25.0–353.0
Glucose (mmol/L)*	4.6 ± 0.2^a^	4.8 ± 0.1^a^	3.7 ± 0.2^b^	4.7 ± 0.2^a^	<0.001	3.1–6.3

Data is shown as Mean ± SEM or Median (range); n = 8 dogs. Different letters within a row indicate significant differences among diets using Tukey’s post-hoc analysis (p < 0.05) after significant p-values reported in one-way repeated measures ANOVA* and Friedman’s one-way ANOVA on ranked data**.

### Sulphur-containing amino acids in plasma

After 28 days of feeding each diet, plasma levels of taurine observed in the dogs were not significantly different among diets (Table 8). Plasma levels of cysteine did vary in dogs after consuming the different diets (Table 8). After consuming the rice or lentil diets, plasma levels of cystine in the dogs were significantly higher than after dogs ate the commercial diet (Table 8). Plasma methionine levels were significantly higher after dogs consumed the commercial diet compared to after the rice or lentil diets (Table 8).

**Table 8:** Plasma sulphur amino acids. Levels of taurine, cystine, cysteine and methionine observed in dogs fed different grain-containing and grain-free diets for 28 days. Diets ordered from left to right in order of increasing total high molecular weight dietary fiber (HMWDF) content.

	Commercial Diet	Rice Diet	Lentil Diet	Wrinkled Pea Diet	p-Value
Cysteine **	0.65 (0.57–0.77)^a^	0.91 (0.74–1.21)^b^	0.86 (0.67–1.12)^b^	0.89 (0.62–1.31)^b^	0.002
Cystine *	11.88 ± 1.01^a^	17.03 ± 1.28^b^	18.50 ± 2.07^b^	15.11 ± 1.32^a,b^	0.004
Methionine *	53.90 ± 2.62^a^	47.71 ± 2.20^b^	45.05 ± 1.53^b^	49.12 ± 1.66^a,b^	0.002
Taurine *	81.21 ± 9.56	87.88 ± 8.79	88.56 ± 13.23	83.62 ± 13.77	0.860

All values are expressed as nmol/mL.

Data is shown as Mean ± SEM or Median (range); n = 8 dogs. Different letters within a row indicate significant differences among diets using Tukey’s post-hoc analysis (p < 0.05) after significant p-values reported in one-way repeated measures ANOVA* and Friedman’s one-way ANOVA on ranked data**.

### NT-ProBNP and cardiac troponin I in plasma

**Fig 2:**
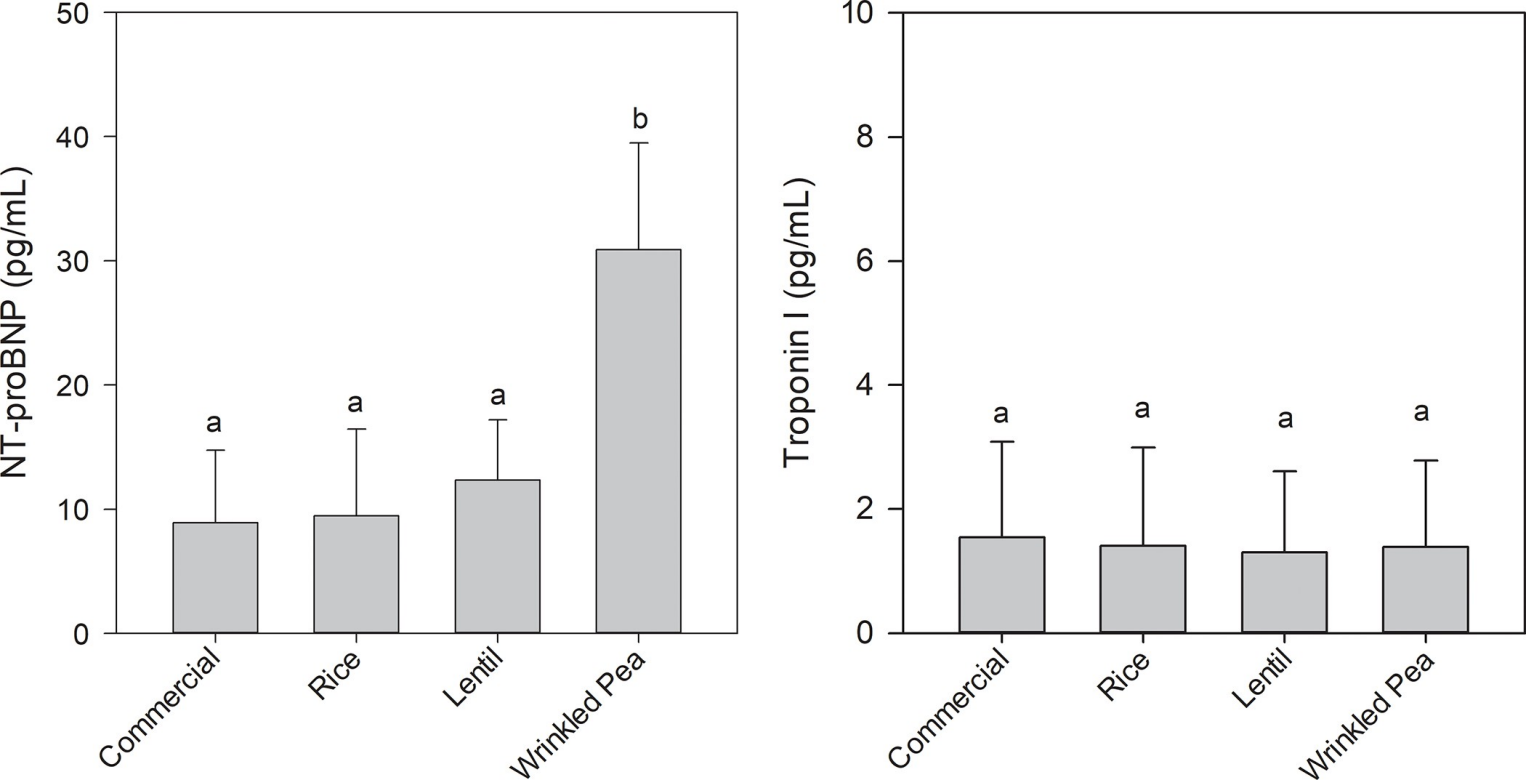
Plasma levels of canine NT-ProBNP (left graph) and cardiac-specific Troponin I (right graph) circulating in dogs after 28 days of feeding each test diet. Diets ordered from left to right in order of increasing total high molecular weight dietary fiber (HMWDF) content. Data is shown as Mean ± SEM; n = 8 dogs. Different letters indicate significant differences using Tukey’s post-hoc analysis (p < 0.05) after conducting a one-way ANOVA.

A diet-related difference in plasma levels of canine NT-ProBNP was detected in dogs after consuming test diets for 28 days. Specifically, levels were significantly higher after dogs consumed the wrinkled pea diet compared to after all other diets (Fig 2). In contrast, the plasma canine cardiac troponin I consistently fell below detection limits in 7 of 8 dogs after consuming all diets. Thus, plasma levels of cardiac troponin did not significantly differ among diets after 28 days consumption (Fig 2).

### Blood pressure and echocardiography

As shown in Table 9, systolic pressure and pulse pressure in dogs were not significantly different after eating grain-containing or pulse-inclusive diets for 28 days. Significant differences were seen in diastolic pressure overall among diets with wrinkled pea diet being the lowest, but pair-wise comparison failed to detect specific differences (Table 9).

**Table 9:** Blood pressure. Systolic, diastolic and pulse pressure measurements observed in dogs fed different grain-containing and grain-free diets for 28 days. Diets are ordered from left to right in order of increasing total high molecular weight dietary fiber (HMWDF) content.

	Commercial Diet	Rice Diet	Lentil Diet	Wrinkled Pea Diet	p-Value
Systolic *	137 ± 6	136 ± 3	138 ± 6	137 ± 6	0.990
Diastolic **	72 (57–102)^a^	80 (68–88)^a^	81 (71–118)^a^	67 (56–112)^a^	0.047
Pulse Pressure *	63 ± 5	58 ± 3	52 ± 5	65 ± 3	0.240

All values are expressed as mmHg.

Data is shown as Mean ± SEM or Median (range); n = 8 dogs. Different letters within a row indicate significant differences among diets using Tukey’s post-hoc analysis (p < 0.05) after significant p-values reported in one-way repeated measures ANOVA* and Friedman’s one-way ANOVA on ranked data**.

Echocardiography values are displayed in Table 10. Heart rate taken simultaneous with echocardiography measurements was significantly lower in dogs after the wrinkled pea diet feeding period (Table 10), but all values remained within the reference range of a healthy adult dog. M-mode demonstrated that left ventricular internal diameter (LVID) at systole was significantly increased in dogs after consuming the wrinkled pea diet for 28 days compared to the comnmercial diet (Table 10).

**Table 10:** Cardiac ultrasound. Echocardiography in dogs fed different grain-containing and grain-free diets for 28 days Diets ordered from left to right in order of increasing total high molecular weight dietary fiber (HMWDF) content.

	Commercial Diet	Rice Diet	Lentil Diet	Wrinkled Pea Diet	p-Value	Reference Interval
**Electrocardiogram**						
Heart Rate (bpm)*	105 ± 12^a,b^	93 ± 5^a,b^	107 ± 8^a^	73 ± 7^b^	0.026	60-120^i^
**M-Mode**						
LVID Systolic (cm/Kg^0.387^)*	0.65 ± 0.03^a^	0.73 ± 0.02^a,b^	0.67 ± 0.03^a,b^	0.77 ± 0.04^b^	0.035	0.50–0.92^ii^
LVID Diastolic (cm/Kg^0.299^)*	1.26 ± 0.04	1.30 ± 0.04	1.24 ± 0.02	1.34 ± 0.05	0.260	1.19–1.63^ii^
**Biplane (Simpson’s Rule)**						
Cardiac Output (mL/min/Kg)*	155.6 ± 28.6^a^	119.7 ± 12.9^a,b^	122.0 ± 16.8^a,b^	79.5 ± 7.7^b^	0.011	118-194^iii^
Stroke Volume (mL/Kg)*	1.46 ± 0.15^a^	1.27 ± 0.11^a,b^	1.15 ± 0.12^b^	1.13 ± 0.07^b^	0.017	1.8–2.1^iii^
Ejection Fraction (%)*	64.3 ± 2.6	66.7 ± 2.2	68.6 ± 2.1	66.1 ± 1.5	0.470	40-72^iv^
Left Ventricular End Systolic Volume (mL/Kg)*	0.81 ± 0.09^a^	0.61 ± 0.03^b^	0.50 ± 0.03^b^	0.57 ± 0.03^b^	0.002	0.35–1.57^iv^
Left Ventricular End Diastolic Volume (mL/Kg)**	2.07 (1.71–3.70)^a^	1.84 (1.39–2.32)^a,b^	1.53 (1.18–2.14)^b^	1.67 (1.37–2.03)^b^	0.006	1.25–3.21^iv^

Data is shown as Mean ± SEM or Median (range); n = 8 dogs. Different letters within a row indicate significant differences among diets using Tukey’s post-hoc analysis (p < 0.05) after significant p-values reported in one-way repeated measures ANOVA* and Friedman’s one-way ANOVA on ranked data**.

Reference Intervals taken from ^i^[17], ^ii^[25]; ^iii^[28]; ^iv^[26]

Left Ventricular Internal Diameter (LVID). N.D. = not determined

Using biplane measurements (Simpson’s rule) of left ventricular volume demonstrated significant differences in cardiac output after consuming different diets. The commercial diet produced the highest cardiac output, while the wrinkled pea diet had the lowest cardiac output, but did not differ from that of the lentil or rice diets (Table 10). Stroke volume was lowest in dogs after feeding the lentil and wrinkled pea diets compared to the commercial diet that had the highest stroke volume (Table 10). However, ejection fraction was unchanged among diets (Table 10). In addition, significant differences were noted among the diets for end-systolic and end-diastolic volumes. In this case, however, the commercial diet produced the highest systolic and diastolic volumes compared to the rice, lentil and wrinkled pea diets in the dogs.

## Discussion

Important findings of this study were that the wrinkled pea diet, but not the lentil diet, caused changes that would suggest early, subclinical stages of DCM after 28 days of feeding in healthy laboratory Beagles. These findings agree with a recent foodomics study of the top 16 dog foods reported by the FDA as being associated with canine nutritional DCM cases, where peas were by far the most common ingredient in culprit diets, less so than lentils [7]. Overall, this study suggests a possible relationship to dietary amylose and oligosaccharides, not soluble or insoluble HMWDF, in diets that may be linked to nutritional DCM in dogs and urgently needs further exploration.

### Properties of diets

The test diets created for this study were formulated to be reasonably isonitrogenous and isocaloric and this was achieved for the lab-made diets. The commercial diet included to provide ‘baseline’ comparisons from the pre-trial period, had comparable protein content, but had noticeably higher energy content. All diets surpassed the minimum requirement set by AAFCO of 18% dietary protein for adult dogs [29]. All test diets were also formulated to meet or just exceed the AAFCO requirements of 0.33% methionine and 0.65% cystine+methionine [29] based on book values of ingredients used. However, analysis of the final extruded and fat-coated product showed some diets failed to meet this recommendation and were slightly below what was required. While this was not intended, these diets are not to be used for clinical or commercial formulations. Moreover, the lower amino acid levels may have facilitated development of adverse effects in the short 28-day feeding period in a dog breed not known to be genetically susceptible to DCM.

One of the primary objectives of this study was to vary the carbohydrate source and fiber type while maintaining approximately 15% enzymatic starch. However, the formulated research diets had almost ten times higher levels of crude fiber than the commercial diet, attributable to the addition of powdered cellulose to produce balanced diets and maintain 15% enzymatic starch across all test diets (Table B of S1 Table). Since we were interested in the role of fiber, the diets showed a good range in insoluble HMWDF, total HMWDF, oligosaccharide and amylose content. Amylose and oligosaccharide content was highest in the wrinkled pea diet, which generally undergoes fermentation within the large intestine [30]. The oligosaccharide content is high in peas and were among the larger group of compounds that distinguished between diets associated or not associated with nutritional canine DCM [7]. This agrees with the findings of this study where the wrinkled pea diet had the highest total oligosaccharide content and was the only diet with detectable raffinose. As reported by Newberry et al. [31] resistant starches, such as amylose contributed to increased dietary fiber, which may be why the pulse diets in this study have higher levels of insoluble HMWDF and total HMWDF. However, dietary cellulose was also a contributing ingredient that influenced the dietary fiber content of our test diets [32].

These pulse flours provided the lentil and wrinkled pea diets with large amounts of plant proteins which decreased the requirements for the use of animal protein (chicken meal). It is known that plant products do not contain taurine, while animal proteins such as seafood and poultry products contain high concentrations of taurine [33]. Despite reduced levels of animal protein in the lentil and wrinkled pea diets, no major differences were observed among diets regarding their taurine levels, due to the appropriate inclusion of both chicken meal and fish meal. Dietary levels of both methionine and cystine+methionine in both pulse-inclusive diets however were lower than the rice and commercial diets. Limited levels of these amino acids in the pulse-inclusive diets were likely due to pulse proteins being limited in sulphur-containing amino acids [3, 34].

### Effect of grain-containing vs grain-free diets on NT-ProBNP, cardiac troponin I and cardiovascular health

In dogs, primary DCM is commonly seen within larger breeds, while Beagles are considered less susceptible [35]. In this study, all plasma cardiac troponin levels were below the sensitivity of the assay in 7 out of 8 dogs after feeding all diets and troponin levels did not differ statistically among diets. The one dog with consistently detectable plasma cardiac troponin levels in this study had levels that were well below that associated with clinical cardiac damage [36]. However, it should be noted that while the current study and previous studies reporting canine reference ranges used immunoassay-based methods to quantitate plasma cardiac troponin and NT-ProBNP, the kits were from different companies [37, 38]. Thus, it is possible that different kits may have led to discrepancies in ranges reported. Nonetheless, relative changes within a given dog in the current study would be robust. Plasma levels of NT-ProBNP was increased after feeding the wrinkled pea diet compared to all other diets. This is a key observation because increased plasma NT-ProBNP along with increased LV chamber diameter and decreased systolic function are indicators of DCM. While this study did not examine reversibility, these adverse cardiac and NT-ProBNP changes may be reversible based on previous studies where dogs with more advanced heart failure previously fed pulse-inclusive diets had improved cardiac function after switching to diets that did not include pulses or potatoes [13, 14].

In dogs with DCM that has advanced to heart failure, tachycardia can be observed, otherwise heart rate may be unchanged with DCM [35]. In the current study, heart rate was instead notably lower in dogs after the wrinkled pea diet feeding period. The cause of this unexpectedly lower heart rate is unclear, but appeared to be a big contributor to the lower cardiac output in dogs after consuming the pea diet [39]. After feeding the wrinkled pea diet, ejection fraction in dogs was also slightly reduced but remained above 60%, while left ventricular internal diameter (LVID) during systole was increased. While these observations of cardiac changes in dogs consuming the wrinkled pea diet are all consistent with early development of DCM, overt DCM is when ventricular ejection fraction falls below 40%, which was not observed in this study [40]. Only a few previous feeding studies examining pulse-inclusive diets have been conducted in healthy dogs or in breeds not known to be susceptible to DCM. Of these, several reported no effect on echocardiography or blood indicators of DCM after feeding laboratory Beagles pea- or fava bean-based diets for only 7 days [21, 41] or faba bean based diets for 28-days [41, 42]. Also, a clinical trial where a similarly high pea-based diet (60% as fed) was fed for 12-weeks to client-owned dogs of variable breeds reported increased LV diastolic dimensions without a change in stroke volume or cardiac output [16]. Thus, the results of the current study agree well with these previous studies in non-susceptible dog breeds where either a longer feeding period of at least a month combined with high inclusion of peas may be necessary to elicit more overt DCM-like changes.

### Effect of grain-containing vs grain-free diets on plasma levels of sulphur-containing amino acids

Taurine is not considered to be essential in dogs as they are able to synthesize taurine from the sulphur-containing amino acids cysteine and methionine [18]. In this study, no major changes in taurine status were observed in the dogs after consuming each diet for 28 days and average values remained within reference range. This agrees with a recent study showing Beagles were resistant to changes in blood taurine levels, while larger, mixed breed dogs showed decreased blood taurine when dietary sulphur amino acids were low for 36 weeks [43]. This latter study also showed that skeletal muscle taurine levels (as a proxy for cardiac muscle) and urinary taurine excretion decreased in both large and small dog sizes while blood levels did not [43]. This suggests that plasma and whole blood taurine may not be good indicators of taurine status but further studies are needed.

Pulses are known to have limiting amounts of cysteine [3]. However, all three lab-made diets (rice, lentil and pea-based) in this study unexpectedly increased plasma levels of cysteine or its reduced form, cystine (after the rice and lentil diets), when compared to the commercial diet in this study. An explanation for this could be attributed to the utilization of plasma methionine as a precursor for both cysteine and taurine in dogs [44]. Methionine-sparing is expected when dietary cysteine is high, but in fact dietary cysteine was low in the pulse-based diets in this study. All plasma methionine values observed in this study fell below the reported reference range by Delaney et al. [45], which was 57.0 nmol/mL. This was true after consuming all lab-made test diets, either as averaged group values or individual dog values, while values after the commercial diet were all within reference range. Comparisons between studies of absolute methionine levels should be interpreted with caution since different methodologies were used. Methionine levels and availability in dogs is largely influenced by taurine and cysteine status [46] or diets with high plant protein content [33]. Beagles are not pre-disposed to either taurine deficiencies or DCM, but have been induced to develop both in an experimental situation [19, 43, 44, 47]. Additional studies comparing these results to the interconnection between sulphur amino acids versus taurine in large, susceptible breeds as well as using skeletal muscle are warranted.

### Effect of dietary fiber and amylose on fecal bile acids

Excretion of total fecal bile acids in this study after feeding each diet for 28 days decreased as dietary insoluble HMWDF, oligosaccharides and amylose increased. For example, the wrinkled pea diet, with the highest level of oligosaccharides, amylose and insoluble HMWDF had the lowest excretion of total fecal bile acids in dogs. This was opposite of what was hypothesized, but it does agree with a 7-day feeding study performed previously by our lab [21]. A large proportion of studies confirm and support that dietary fiber is able to bind bile acids in the intestinal lumen, which in turn would lead to an increased excretion of total fecal bile acids [48–50]. Dogs have been hypothesized to be susceptible to taurine loss because taurocholate is the predominant bile salt in dogs [18]. Soluble dietary fiber would bind the taurocholate, preventing reabsorption into the entero-hepatic circulation and depletion of taurine [18]. Despite high soluble fiber levels in the pea diet in this study, results instead agreed with that from a study performed by Pezzali et al. [51] where pulse-inclusive diets failed to increase excretion of fecal bile acids in dogs. Furthermore, a recent meta-analysis demonstrated that high levels of carbohydrates are instead associated with decreased excretions of fecal bile acids [51]. One potential link between taurine and fiber that has some experimental support comes from the effect of fiber, particularly oligosaccharides, to increase intestinal microbiome mass and microbial metabolism of taurine before it is absorbed by the dog [43]. While this study showed that wrinkled pea diet was the only diet with detectable raffinose, both verbascose and stachyose were also higher than the lentil diet. Thus, the specific oligosaccharide responsible could not be identified. Overall, more studies should be performed to further investigate which fiber components most closely connect to the microbiome, microbial taurine metabolism and dietary taurine availability in dogs.

### Macronutrient and amino acid total tract apparent digestibility

Consistent with a 7-day study conducted in our lab with similar diets, the results of this 28-day study found that pulse-based diets decrease the digestibility of some macronutrients [21]. An explanation for this could be due to the increased amounts of dietary amylose and fiber found in the diets that is known to influence nutrient digestibility [52]. Crude protein digestibility was lower in the two pulse-containing diets compared to rice diet. This could be due to the higher resistant starch content of pulses since a previous dog study reported similar decreased protein digestibility with graded increases in resistant starch [53]. Another reason for this could be due to the rice diet having higher amounts of more digestible animal proteins, while the pulse-based diets contained less animal protein and higher amounts of plant protein. Another possible explanation for limited protein digestibility is due to the intrinsic interactions of amylose [54]. Decreased starch digestibility in this study with the wrinkled pea diet could have been due to the higher temperature needed to gelatinize high amylose starch and lower gelatinization. Alternatively, interactions between starch and lipids during extrusion could have further reduced starch digestibility of the kibble [5, 55]. In addition to this, lipid digestibility was also reduced in the pea diet compared to the rice diet and could be due to the higher amylose and dietary fiber content in peas. Digestibility of methionine and cystine+methionine in this study were also lower in the lentil and wrinkled pea diets when compared to the rice diet. It is known that sulphur-containing amino acid excretion can increase with increasing levels of dietary fiber in both dog and cats [50, 56]. However, this study was unable to determine which fiber fraction or if amylose caused digestibility decreases in the different diets. In addition to this, no major decreases in the digestibility of taurine were observed, with all values close to 100% in dogs among all diets after 28 days of feeding. An explanation for this could from a cat study where unabsorbed taurine in the gastrointestinal tract is degraded by microbial populations whose numbers are increased after feeding diets with high amounts of fermentable fiber [57] or that taurine is a nutrient that is rapidly, completely absorbed under all circumstances in dogs.

### Effect of diet on blood chemistry and complete cell counts

In this study, there are no major findings demonstrating compromised overall health in dogs consuming both grain-containing and pulse-inclusive diets. Following the commercial diet feeding period, cholesterol levels in the dogs were elevated compared to the other diets, but all values were within reference range. The commercial diet had much lower crude fiber, oligosaccharides and total HMWDF levels than any of the test diets in this study. Cholesterol is thought to bind to fiber, ultimately leading to a fecal clearance [58] and is consistent with findings in this study. Blood glucose levels were also higher in diets with increased levels of amylose, which is suspected to be due to a gluconeogenic effect. Total bilirubin and indirect bilirubin were also seen to decrease in the pulse-inclusive diets, the cause for this however remains unknown. The current study in Beagles observed increases in serum phosphorus after feeding the lentil-based diet which agrees with a previous 28-day study in adult Labrador retrievers fed a diet high in both peas and lentils as well as 7-and 28-day studies in Beagles fed a commercial pulse-containing diet [15, 41, 42]. However, in the current study RBC counts were similar after feeding all three lab-made diets. Where a difference in RBCs was observed in the current study was between the commercial diet compared to the rice and pea diets. The lack of effect of pulse-based diets on RBCs agrees with a previous 28-day feeding study in Beagles from this group where a high protein pea-containing commercial diet had no effect on RBCs compared to a commercial diet with no pulses [42]. This differs from the previous Labrador retriever study that observed anemia after feeding pulse-based diets [15] as well as our previous 7-day study in Beagles using a high protein, pea-based commercial diet where RBCs were within all normal range but lower compared to a pulse-exclusive commercial diet. One would expect that a longer feeding period should have produced a greater effect on RBCs if it is diet-related. In contrast, the two previous studies from our group show only a transient RBC decrease, suggesting instead that this was either random or due to other unidentified factors.

### Study strengths & limitations

A strength of this study was the use of two grain-containing and two pulse-inclusive diets to examine how they impact cardiovascular function in dogs after feeding each diet for a longer term (28 days) than a previous study from this group [21]. Moreover, a strength is the use of two separate rice feeding periods that produced statistically similar results, lending confidence to the repeatability of all results. Another strength included examining the impact pulse-inclusive diets have on plasma levels of sulphur-containing amino acids and taurine. In addition, this study contains blood chemistries and CBC analyses in dogs after feeding grain-containing and pulse-inclusive diets, which has sparsely been reported in current literature.

A major limitation was the formulation of the test diets to include 15% enzymatic starch, which required 58% inclusion of wrinkled pea flour to achieve. However, this level of pea flour inclusion is comparable to some commercial vegan dog foods [16]. This extremely high inclusion of pulse flour is responsible for the low animal protein and low methionine levels in the diets. Related to this, a relative lack of effect of the lentil diet in this study may simply be to its lower inclusion level (42%) than the wrinkled pea. Higher inclusion of lentil flour may have produced similar results to the pea diet. While these test diets may not be representative of most commercial diets, we did include data from a popular grain-containing commercial diet used during the pre-trial phase as a baseline comparison, albeit with a different nutrient profile from the test diets. Beagles are not predisposed to primary DCM or taurine deficiencies, but if we managed to observe DCM-like changes in this resistant breed, then it seems likely that more susceptible breeds would have suffered greater adverse cardiac changes. Another limitation of this study was the small sample size of eight beagles, due to budget and housing constraints. Ideally, larger scale prospective studies using client-owned dogs from multiple breeds fed controlled diets would strengthen conclusions. However, ethical constraints likely preclude this type of study if pets are likely to develop DCM. Another limitation is use of apparent total tract digestibility instead of ileal cannulation to determine true digestibility. Apparent total tract digestibility is minimally invasive, needed to satisfy ethical review and allow research dogs to be adopted into homes when retired from nutrition studies. Another limitation was the ELISA kits used to detect both markers of cardiac damage and stretch. While both the NT-proBNP and hs-cTnI kits used canine-specific antibodies, we did not perform an independent confirmation of the manufacturer’s claim of specificity and both kits were not sensitive enough to detect values in all samples. Thus, there is the possibility that we may have missed a smaller change in one or both of these markers. However, these issues do not take away confidence in the conclusion that feeding the wrinkled pea diet for 28 days increased NT-proBNP levels. A final limitation is that blood pressure was measured using an oscillometric blood pressure cuff which is a relatively insensitive method of detection compared to intra-arterial measurement. Thus, small changes in blood pressure would not have been detectable, but we can say with confidence that any change missed would have been subclinical.

## Conclusions

After 28 days of feeding test diets in Beagles, this study found that a high amylose, high HMWDF and verbascose-containing wrinkled pea diet increased left ventricular diameter at systole and decreased stroke volume, along with increased plasma NT-ProBNP, albeit in a sub-clinical manner. These changes were not associated with changes in plasma taurine levels. The lentil diet, another grain-free diet tested, did not produce DCM-associated changes, compared to the rice diet or the commercial diet, another grain-containing diet. Whether longer feeding periods than 28 days with the wrinkled pea diet would have produced overt DCM or if the lentil diet would eventually produce similar changes, particularly if inclusions was higher, is unclear. Moreover, aside from an increase in serum phosphorus after feeding the lentil diet, pulse-inclusive diets did not have any major detrimental impacts on overall health that was observed through blood chemistries and CBCs. While plasma taurine was unchanged with all diets, plasma methionine was similarly lower for all lab-made diets compared to the commercial diet. The pulse-inclusive diets were seen to have higher levels of amylose, total HMWDF and oligosaccharides, which in turn decreased macronutrient and amino acid apparent digestibility, but no effect on taurine. Higher content of dietary fiber produced decreases fecal bile salt excretion after 28 days, similar to what we observed previously after 7 days. Due to the limitations of having all diets formulated to 15% enzymatic starch, high-fiber pulse diets contained less animal-sourced protein and higher plant-based protein, while the high-fiber rice diet had more animal-sourced protein. This could be an important factor leading to the decreases in nutrient digestibility in the pulse-inclusive diets. Overall, there is further evidence to support a link between DCM-like changes and the wrinkled pea diet, but not lentil diet in a small dog breed that is not known to be susceptible to DCM. Further longer term studies are urgently needed to better explore these potential links between specific dietary factors and nutritionally-mediated canine DCM.

## Supporting information

S1 Table Contains supporting tables. (DOCX)

S1 Text Raw data from Quilliam et al. study. (XLSX)
