# Correction: Effects of a 28-day feeding trial of grain-containing versus pulse-based diets on cardiac function, taurine levels and digestibility in domestic dogs

**DOI:** 10.1371/journal.pone.0308509

**Published:** 2024-08-02

**Authors:** Chloe Quilliam, Luciana G. Reis, Yikai Ren, Yongfeng Ai, Lynn P. Weber

There are errors in the Funding statement. The correct Funding statement is as follows: Funding for this study was provided by the Agriculture and Agri-Food Canada’s AgriScience Program (Canadian Pulse Science Research Cluster) under the Canadian Agricultural Partnership (Project Number: ASC-06). Additional funding also came from a Natural Sciences and Engineering Research Council (NSERC) Discovery grant held by LW (RGPIN 05812–2016). In-kind donation of some feed ingredients was provided by Horizon Pet Foods (Rosthern, SK Canada). Lentil seeds and wrinkled pea flour were kindly provided by T Warkentin (Plant Sciences, University of Saskatchewan, Saskatoon, SK Canada) and Alliance Grain Traders (Saskatoon, SK Canada), respectively. The wrinkled pea flour is not a commercially available ingredient. The funders had no role in study design, data collection and analysis, decision to publish, or preparation of the manuscript.

There are errors in the Competing Interests statement. The correct Competing Interests statement is as follows: The authors declare that this study received funding or in-kind support from the Agriculture and Agri-Food Canada’s AgriScience Program (Canadian Pulse Science Research Cluster) under the Canadian Agricultural Partnership, Natural Sciences and Engineering Research Council (NSERC), Alliance Grain Traders (Saskatoon, SK Canada), Horizon Pet Foods (Rosthern, SK Canada) and Dr. Tom Warkentin (University of Saskatchewan, SK). The funders were not involved in the study design, collection, analysis, interpretation of data, or the writing of this article or the decision to submit it for publication.
