# Supplementary material for: Effects of a 28-day feeding trial of grain-containing versus pulse-based diets on cardiac function, taurine levels and digestibility in domestic dogs
Source: PLoS One. 2023 May 25;18(5):e0285381. doi: 10.1371/journal.pone.0285381 (PMC10212094; doi:10.1371/journal.pone.0285381)
Supplement: S1 Table — (DOCX) [file pone.0285381.s001.docx]

# **S1 Tables: Supporting Information**

| Flour | Starch content (%, dry flour basis) *^c^* | Amylose content (%, dry flour basis) *^d^* | Amylose content (%, dry starch basis) *^e^* | Protein content (%, dry flour basis) *^f^* | Crude fiber content (%, dry flour basis) *^g^* | Fat content (%, dry flour basis) *^h^* | Ash content (%, dry flour basis) *^i^* | Dry matter (%) |
| --- | --- | --- | --- | --- | --- | --- | --- | --- |
| Rice | 86.5 ± 0.5 ^a^ | 21.9 ± 0.4 ^a^ | 25.3 ± 0.5 ^a^ | 7.3 ± 0.1 ^a^ | 0.02 ± 0.01 ^a^ | 0.74 ± 0.07 ^a^ | 0.48 ± 0.02 ^a^ | 88.1 ± 0.0 |
| Lentil | 47.4 ± 0.3 ^b^ | 17.4 ± 0.4 ^b^ | 36.8 ± 0.8 ^b^ | 27.6 ± 0.1 ^b^ | 3.55 ± 0.06 ^b^ | 0.39 ± 0.05 ^a^ | 2.41 ± 0.01 ^b^ | 93.6 ± 0.1 |
| Wrinkled pea | 34.4 ± 0.3 ^c^ | 27.1 ± 0.2 ^c^ | 78.8 ± 0.6 ^c^ | 26.4 ± 0.2 ^c^ | 7.00 ± 0.17 ^c^ | 1.90 ± 0.06 ^b^ | 3.40 ± 0.02 ^c^ | 92.8 ± 0.0 |

S1 Table A. Chemical compositions of flour samples *^a, b^*

*^a^* Values are presented as mean ± standard error of the mean of triplicate measurements.

*^b^* Values followed by the same letter in the same column are not significantly different at *p* < 0.05.

*^c^* Determined using Megazyme Total Starch Assay Kit following AACC Method 76-13.01.

*^d^* Determined using an iodine colorimetric method of Chrastil (1987).

*^e^* Amylose content (%, dry starch basis) = [Amylose content (%), dry flour basis] / [Total starch content (%), dry flour basis] × 100.

*^f^* Determined using a Nitrogen/Protein Analyzer (CN628, LECO Corporation, St. Joseph, MI, USA), with a conversion factor of 6.25.

*^g^* Determined by Central Testing Laboratory Ltd. (Winnipeg, Manitoba, Canada), following Crude Fiber Method by Ankom Technology (2017).

*^h^* Determined by Central Testing Laboratory Ltd. (Winnipeg, Manitoba, Canada), following AOCS Method Am 5-04.

*^i^* Determined by Central Testing Laboratory Ltd. (Winnipeg, Manitoba, Canada), following AOAC Method 942.05.

S1 Table B: Formulation of rice, lentil and wrinkled pea diets. Diets ordered from left to right in order of increasing total fiber content.

|  | Rice Diet | Lentil Diet | Wrinkled Pea Diet |
| --- | --- | --- | --- |
| Flour | 23.2 | 42.2 | 58.1 |
| Chicken By-Product Meal | 39.3 | 23.1 | 14.4 |
| Cellulose | 15 | 12.2 | 5 |
| Chicken Fat | 10 | 10 | 10 |
| Fish Meal | 5 | 5 | 5 |
| Canola Oil | 5 | 5 | 5 |
| Celite | 1 | 1 | 1 |
| Vitamin/Mineral Premix* | 1 | 1 | 1 |
| Sodium Chloride | 0.3 | 0.3 | 0.3 |
| Choline Chloride | 0.1 | 0.1 | 0.1 |
| Calcium Carbonate | 0.05 | 0.05 | 0.05 |
| Dicalcium Phosphate | 0.05 | 0.05 | 0.05 |

All values are expressed as % inclusion as fed. *Included addition of antioxidant Naturox (Kemin, Des Moines, IO USA)

S1 Table C: Extruder parameters used to create test diets

| Parameters | Condition |
| --- | --- |
| Feed Moisture (%) | 25 |
| Solid Feed Rate (kg/h) | 10 |
| Screw Speed (rpm) | 300 |
| Temperature (Section 1, ˚C) | 30 |
| Temperature (Section 2, ˚C) | 60 |
| Temperature (Section 3, ˚C) | 90 |
| Temperature (Section 4, ˚C) | 120 |
| Temperature (Section 5, ˚C) | 120 |
| Temperature (Section 6, ˚C) | 120 |
| Die Diameter (mm) | 4 |

S1 Table D: Ingredient list of commercial diet fed in the pre-trial phase of the study

|  | Ingredients: |
| --- | --- |
| Commercial Diet | Chicken, Brewers Rice, Whole Grain Wheat, Poultry By-Product Meal (Natural Source of Glucosamine), Corn Gluten Meal, Whole Grain Corn, Animal Fat (Preserved with Mixed-Tocopherols, Form of Vitamin E), Corn Germ Meal, Fish Meal (Natural Source of Glucosamine), Animal Digest, Dried Egg Product, Salt, Potassium Chloride, Calcium phosphate, Calcium Carbonate, Vitamin E Supplement, Choline Chloride, Zinc Sulfate, Ferrous Sulfate, L-Ascorbyl-2-Polyphosphate (Source of Vitamin C), L-Lysine Monohydrochloride, Manganese Sulfate, Niacin, Vitamin A Supplement, Calcium Pantothenate, Thiamine Mononitrate, Copper Sulfate, Riboflavin Supplement, Vitamin B-12 Supplement, Pyridoxine Hydrochloride, Garlic Oil, Folic Acid, Vitamin D-3 Supplement, Calcium Iodate, Biotin, Menadione Sodium Bisulfite Complex (Source of Vitamin K Activity, Sodium Selenite |

S1 Table E: Variability in select echocardiography measurements from the current study. Each echocardiography session was performed in duplicate on each dog for the rice diet, with duplicate feeding periods of 28-days each performed during this experiment for a total of 4 measurements for each dog, all performed by the same sonographer. Values are show as coefficient of variation (%).

| Echocardiography Measurement | Variability |
| --- | --- |
| End-diastolic volume | 14.3 |
| End-systolic volume | 14.1 |
| Stroke volume | 19.0 |
| Ejection Fraction | 10.1 |
